# Optimized NFC Circuit and Coil Design for Wireless Power Transfer with 2D Free-Positioning and Low Load Sensibility †

**DOI:** 10.3390/s21238074

**Published:** 2021-12-02

**Authors:** Guilherme Germano Buchmeier, Alexandru Takacs, Daniela Dragomirescu, Juvenal Alarcon Ramos, Amaia Fortes Montilla

**Affiliations:** 1 Continental Automotive France, 31100 Toulouse, France; juvenal.alarcon.ramos@continental-corporation.com (J.A.R.); amaia.fortes.montilla@continental-corporation.com (A.F.M.); 2 Laboratory for Analysis and Architecture of Systems (LAAS-CNRS), University of Toulouse, CNRS, 31400 Toulouse, France; daniela.dragomirescu@laas.fr

**Keywords:** wireless power transfer, free-positioning, NFC, high-efficiency, load independent

## Abstract

This paper proposes a method for optimizing and designing a wireless power transfer system operating at 13.56 MHz. It can be used as guidelines for designing coils for the new-trending technology that enables NFC devices to not only to communicate but also to charge. Since NFC wireless charging is an emerging technology, it is of interest to propose optimizations and a dedicated circuit design for such systems. This work proposes an optimization procedure to calculate the dimensions of a transmitter and receiver pair that assures the highest efficiency while considering all possible positions of a receiver that is placed on a desired surface. This procedure seeks to facilitate and automate the design of rectangular-shaped coils, whereas the literature proposes mainly square-shaped coils. Afterwards, a circuit analysis was conducted, and the series-parallel compensation network is proposed as the most promising topology of the receiver to assure a low efficiency sensibility to load variations for 13.56 MHz wireless power transfer systems. A pair of optimized transmitter and receiver coils is prototyped, and the experimental results are tested against the theory. The transmitter of 7 cm×11.4 cm and receiver of 4 cm ×4 cm are separated by 10 mm. The receiver can move on a surface of 8 cm ×12 cm and the load can vary from 36 Ω to 300 Ω while assuring a minimum and maximum efficiency of 80% and 88.3%, respectively.

## 1. Introduction

As technology advances, several electronic devices are getting smaller and it turns out to be more difficult to integrate charging connectors in such devices. Therefore, it would be more suitable and flexible if they can have their battery charged wirelessly, for example, electric vehicles, smartphones, key fobs, medical implants, waterproof devices, etc. However, devices that only need to be turned on when placed in the proximity or vicinity of another device do not necessarily need batteries, because they can harvest energy from the other device during the operational time [1]. Such a case is near-field communication (NFC), with which passive devices receive energy from a transmitter and use this energy to operate and to communicate [2]. This background strengthened the wireless power transfer (WPT) and energy harvesting technologies, which have been emerging in many sectors of the industry as an alternative to classical wire charging and batteries.

Inductive power transfer is the most used technique for WPT in short-range distances; that is, up to a few centimeters. It is based on the coupling between the transmitter (Tx) and the receiver (Rx) coils [1]. The most efficient configuration for transferring energy wirelessly occurs when both coils are identical and close to each other. However, in real applications it is rare to have devices with the same coil size.

The consequence of misalignment between coils is that the efficiency of the power transfer drops. In addition, assuming there is a battery charging on the receiver, the impedance changes with time and thus the efficiency also changes. To compensate for the loss of efficiency due to misalignment of the coils, many studies of free-positioning WPT systems have been made [3,4,5]. However, they are based on multiple transmitters, switching impedance matching networks that are manually controlled, or 3D transmitters. Such solutions are either difficult to implement in high frequencies or space-consuming in terms of circuit implementation.

Most of actual wireless chargers are based on the Qi standard, which operates between 87 kHz and 205 kHz and allows a maximum separation of 4 cm [6]. Operating at 13.56 MHz, the NFC allows a bigger separation than the Qi (up to 15 cm [7,8,9]) and even though the technology was firstly created for communication, the NFC Forum [10] has recently published a technical specification for the NFC as a wireless power transfer solution.

This new trend in NFC wireless charging aims to cover efficiently the interval of 0 to 1 W with smaller coils, which can be more easily integrated into small devices. WPT via NFC is a very compact solution, because not only are the coils much smaller than Qi coils but also it would be possible to use the same coil for charging and to make the communication secure. In addition, contrary to NFC wireless charging, Qi charging usually requires a ferrite to boost efficiency, which is expensive and space-consuming. From this point of view, several small smart devices that already have NFC coils could be easily updated to enable wireless charging. Besides, the higher the operation frequency, the higher the efficiency [11], which draws more attention to WPT systems operating at 13.56 MHz than 200 kHz. Given all the advantages of using the NFC as WPT solution, it is a promising solution to assure the free-positioning of the receiver.

The modelling of the NFC coils depends on their shape, dimensions, number of turns, width of the conductor, and the pitch between two adjacent turns. Their electrical parameters are difficult to be theoretically calculated as a function of dimensions; thus, most designs are made directly through electromagnetic simulations and adjusted with retro-simulations, which is very time consuming. Another approach that is commonly used is to use the available space of the device to arbitrarily design the dimensions of the coil [12]. These methods produce functional coils but not optimal coils, allowing the system to operate at maximum efficiency. However, in the literature, some optimization methods are proposed, such as in [12] for circular coils of wire and in [13,14,15] for printed spiral coils (PSCs). However, these references still consider optimization methods for one position of the receiver and the PSCs are square-shaped, whereas rectangular-shaped coils are not treated and would also be of interest.

This work focuses on designing a WPT system operating at 13.56 MHz that can operate at high efficiency regardless of the position of the receiver and its load. A method for numerically calculating the electrical parameters of a rectangular PSCs as function of dimension is proposed. A first approach to this method was made in prior work [16] and is extended upon in this work. This procedure seeks to facilitate and automate the design of high-efficiency coils. In addition, the receiver compensation network is analyzed so it can be selected in such a fashion that the efficiency is less dependent on load variations.

This article is divided as follows. The background theory of a WPT system is introduced in Section 2. The optimized coil design method proposed in the prior study is realized and presented in Section 3, with the experimental results. Afterwards, in Section 4, the proposed optimization method is simplified, and some further analysis are introduced for designing and optimizing a more complete system. The experimental results are presented in Section 5. Finally, Section 6 concludes this work.

## 2. The WPT System Overview

**Figure 1:**
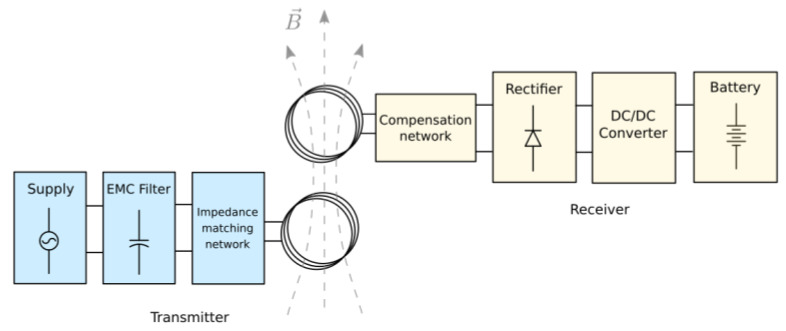
Scheme of a WPT system.

A WPT system is composed by a transmitter (Tx) and receiver (Rx). The Tx is basically composed of a power supply, an EMC filter, an impedance matching network, and a coil. The Rx can be divided as follows. A coil, an impedance compensation network, a bridge rectifier, a voltage regulator, and a load. The nature of the load may vary according to the application, but for WPT systems it is usually a battery. Figure 1 illustrates the scheme of the system.

For embedded systems, the power supply is usually provided by a chip [17], through which a full bridge creates a square wave signal that is usually filtered by an EMC filter to keep only the fundamental frequency. However, it is also possible to use an independent power supply, such as class D and E amplifiers [18,19,20,21], which generates directly a sinusoidal signal that does not necessarily need filtering.

The impedance matching network is crucial to adapt the coil’s impedance to a real value, so the power supply only delivers active power. Otherwise, reactive power is injected into the circuit, decreasing the system’s efficiency, delivering less power [22]. Some approaches to adapt automatically or manually the impedance of a NFC coil are discussed in [23,24], where it can be seen that all solutions present some disadvantages, such as narrowing the range of tunable coils, adding losses due to biasing circuits, or consuming a large surface of circuit implementation.

Both transmitter and receiver coils are coupled magnetically in order to allow energy transfer. The alternating current that flows through the Tx coil generates an alternating magnetic field that traverses the Rx coil and induces a voltage on it. This voltage acts as a power supply to the Rx, delivering power to its load.

Regarding the receiver’s circuit, a capacitive compensation network is used to enable resonance and harvest the maximum energy from the Tx. The rectifier is used to convert the AC signal to DC, which can be used to charge a battery or supply a device. The intermediate block DC–DC converter is used to regulate the DC level in order to adapt the load voltage requirement.

## 3. Coil Design and Optimization of Its Dimensions

This section introduces the rectangular PSC used in this work as well as the mathematical formulation of its electric parameters as a function of dimensions. Afterwards, through an example, the equations are used to optimize the dimensions of a pair of transmitter–receiver coils, assuring a high efficiency for all positions of the receiver over an entire surface. In other words, the optimization method forces the receiver to be able to be freely positioned over the desired surface, assuring the efficiency to be greater than the defined threshold.

### 3.1. Coil Design

**Figure 2:**
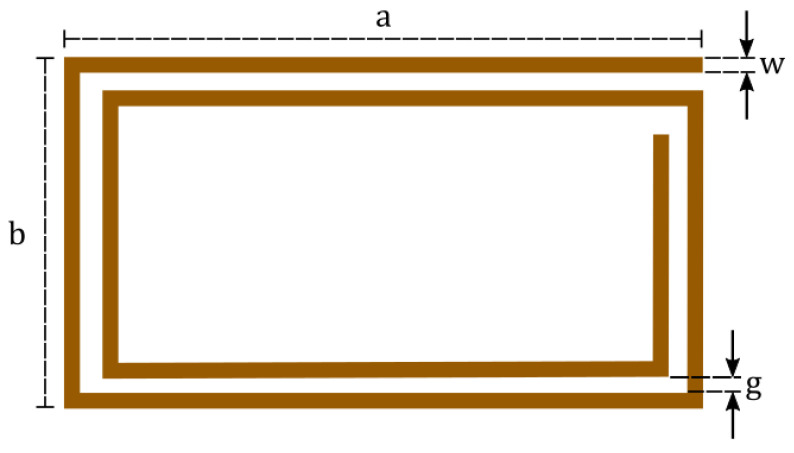
Two-turn rectangular coil with its dimensional parameters.

Seeking to design NFC coils, this work treats and optimizes a rectangular planar coil of rectangular cross-section. Therefore, the coil can be easily manufactured as a PCB. Even though a rectangular shape was selected, the study could be extended to other layouts, such as polygonal or circular. Figure 2 illustrates such a structure with its dimensions. In addition to the figure, two other parameters must be considered: the number of turns N and the conductor thickness t.

**Figure 3:**
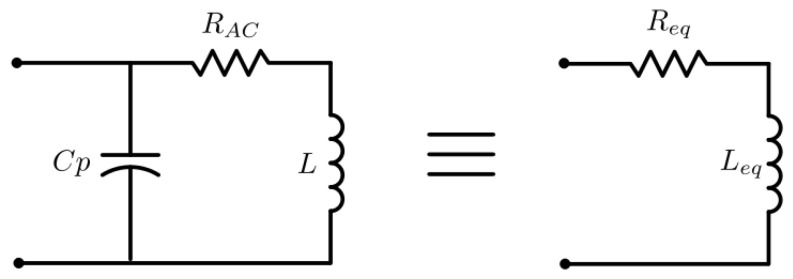
Coil equivalent circuit.

A coil can be represented with an equivalent circuit composed by an inductance *L* with a series resistance *R_AC_* and a capacitor *C_p_* in parallel. Figure 3 illustrates the circuit.

#### 3.1.1. Coil Inductance

The inductance of a rectangular planar coil can be calculated by the contribution of three terms: $L_{0}$, which is the self-inductance of each segment of the coil, and $M_{+}$ and $M_{-}$, which are the sum of the positive and negative mutual inductances between the segments, respectively. The authors in [25,26] proposed a closed formula to calculate these terms, starting by the self-inductance, $L_{0}$, which can be calculated by Equation (1).

(1) $$L_{0}=800N\ell_{avg}\left[\ln\left(\frac{2\ell_{avg}}{w+t}\right)+0.50049+\frac{w+t}{3\ell_{avg}}\right],$$

where $\ell_{avg}=\left(a_{min}+b_{min}\right)/2$ and $a_{min}$ and $b_{min}$ are the internal length and width of the coil, respectively. All parameters are in SI units.

Regarding the mutual inductance terms, approximative closed formulas can be found in [26]. Therefore, to the inductance of the coil can be calculated by

(2) $$L=L_{0}+M_{+}+M_{-}.$$

#### 3.1.2. AC Resistance

Because the coil is made of a finite conductivity conductor, a series DC resistance is added to the inductance. It can be expressed by

(3) $$R_{DC}=\frac{\rho \ell }{wt}\;\;,$$

where ρ is the resistivity and ℓ is the total conductor’s length.

However, at high frequencies, such as 13.56 MHz, the DC resistance is not sufficient to represent the total resistance of a conductor. Therefore, the skin effect must also be considered. Once the cross section of the conductor is rectangular, the AC resistance is dramatically difficult to treat mathematically. Experimental curves are proposed by Haefner [27], but they have no analytical analysis. A very reasonable approximation of the AC resistance is introduced in [28]; the overall equation proposed for rectangular conductors is given by Equation (4).

(4) $$R_{AC}=R_{DC}\frac{\left(1-e^{-0.038\frac{\sqrt{wt}}{\delta }}\right)\left[0.06+0.22\ln\left(\frac{w}{t}\right)+0.28{\left(\frac{t}{w}\right)}^{2}\right]+1}{1-e^{-\left(\frac{2\delta }{t}+\frac{2\delta }{w}\right)}}\;,$$

where

(5) $$\delta =\sqrt{\frac{\rho }{\pi f\mu_{r}\mu_{0}}}$$

is the skin depth computed as function of the operating frequency *f*, resistivity of the conductor ρ, and relative magnetic permeability $\mu_{r}$.

#### 3.1.3. Parasitic Capacitance

When two conductors are in the proximity of each other, they can cross-talk through the parasitic capacitance created between them. The calculation of such a capacitance for a PSC is approximated by the Equation (6) [14], which considers the effect of the substrate over which the conductors are placed.

(6) $$C_{p}\approx \left(0.9\epsilon_{r_{air}}+1.1\epsilon_{r_{subs}}\right)\frac{\epsilon_{0}\ell_{g}t}{g}\;,$$

where $\epsilon_{0}=8.84\times 10^{-12}$ is the vacuum permittivity, $\epsilon_{r_{air}}$ is the dielectric constant of the air between the conductors, $\epsilon_{r_{subs}}$ is the dielectric constant of the substrate (4.4 for FR4), t is the thickness of the conductor, and $\ell_{g}$ is the length of the gap.

For a N-turn rectangular coil, such as the one illustrated in Figure 2, the length of the gap can be calculated by

(7) $$\ell_{g}=\left(N-1\right)\left[2\left(a+b\right)-4Nw-\left(4N-3\right)g\right].$$

It must be noted that because the conductor thickness is usually small for planar coils, the parasitic capacitance tends to be small. However, depending on the inductance value, even small values of capacitance can impact the behavior of the circuit, because the coil’s self-resonance frequency might be close to the operating frequency. This analysis is held in the next subsection.

#### 3.1.4. RL Equivalent Circuit

As Figure 3 suggests, the coil equivalent circuit can be simplified to a series RL circuit, composed by an equivalent series resistance $R_{eq}$ and an equivalent series inductance $L_{eq}$. These two parameters can be expressed by

(8) $$R_{eq}=\frac{R_{AC}}{{\left(\omega R_{AC}C_{p}\right)}^{2}+{\left[1-{\left(\frac{\omega }{\omega_{sr}}\right)}^{2}\right]}^{2}}$$

and

(9) $$X_{eq}=\frac{\omega L\left[1-{\left(\frac{\omega }{\omega_{sr}}\right)}^{2}\right]-\omega R_{AC}^{2}C}{{\left(\omega R_{AC}C_{p}\right)}^{2}+{\left[1-{\left(\frac{\omega }{\omega_{sr}}\right)}^{2}\right]}^{2}},$$

where

(10) $$\omega_{sr}=\frac{1}{\sqrt{LC_{p}}}\;$$

is the self-resonance angular frequency of the coil.

The more the operating frequency is negligible regarding the self-resonance frequency, the more the capacitance effect is negligible. However, if the operating frequency is close to the self-resonation frequency, the equivalent series inductance and resistance may vary. The main concern about this fact is that as the equivalent resistance increases, power losses also increases, which is not desired for high-efficiency requirements.

For PCB coils, the AC resistance varies from a few dozens of mΩ to a few Ω, whereas the parasitic capacitance rarely exceeds few pF. Therefore, the capacitive reactance is much bigger than the AC resistance, and thus the equivalent series resistance can be approximated to Equation (11).

(11) $$R_{eq}\approx \frac{R_{AC}}{{\left[1-{\left(\frac{\omega }{\omega_{sr}}\right)}^{2}\right]}^{2}}\;.$$

Considering a maximum tolerance increase of α percent on the power losses due to operation near to the self-resonance frequency, the following relation can be stablished:

(12) $$\frac{\omega }{\omega_{sr}}<\sqrt{1-\frac{1}{\sqrt{1+0.01\alpha }}}\;.$$

In other words, Equation (12) allows to calculate the minimum self-resonance frequency of a coil for which it will dissipate less than α percent of power when compared to an ideal coil. If Equation (12) is satisfied for a sufficient low α (e.g., α=1%), the parasitic capacitance of the coil can be neglected, because the power losses caused by the capacitance effect would be negligible.

### 3.2. Optimization of Coil Dimensions by Maximizing the Efficiency

In this subsection, an example of coil optimization is described with theoretical, simulation, and experimental results. To do so, first a circuit analysis is made and then the optimization procedure is described.

#### 3.2.1. Circuit Analysis

**Figure 4:**
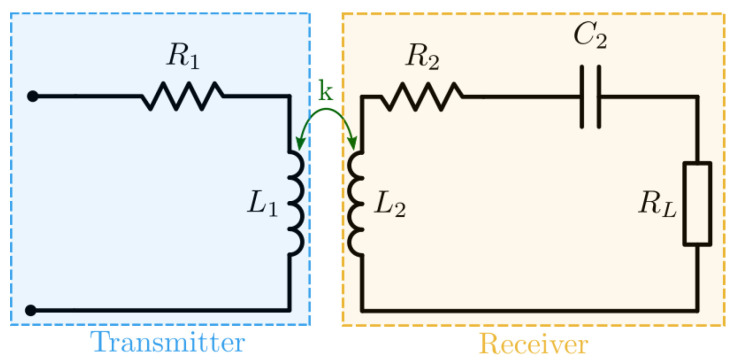
Reference circuit for optimizing the dimensions of the coils.

The circuit of Figure 4 is considered for this optimization example. A capacitance $C_{2}$ is connected in series with the receiver’s coil to increase the system’s efficiency by enabling resonance. Both coils are magnetically coupled with a coupling coefficient k.

The objective is to find the dimensions of the coils that optimize the circuit efficiency, when the receiver is free to move over a given surface. The efficiency is defined by the ratio of the active power delivered to the load and active power injected in the transmitter. It can then be expressed by the following equation:

(13) $$\eta =\frac{\omega^{2}M^{2}R_{L}\;}{R_{1}{\left(R_{2}+R_{L}\right)}^{2}+R_{1}{\left(\omega L_{2}-\frac{1}{\omega C_{2}}\right)}^{2}+\omega^{2}M^{2}\left(R_{2}+R_{L}\right)}\;,$$

where M is the mutual inductance and the relation with the coupling coefficient is the following:

(14) $$M=k\sqrt{L_{1}L_{2}}\;.$$

If the series capacitance resonates with the receiver coil, the denominator’s middle term cancels out, resulting in a higher efficiency, simplifying Equation (13) to:

(15) $$\eta =\frac{\omega^{2}M^{2}R_{L}\;}{R_{1}{\left(R_{2}+R_{L}\right)}^{2}+\omega^{2}M^{2}\left(R_{2}+R_{L}\right)}\;.$$

There is also a load for which the power transfer is maximal, it is defined as follows:

(16) $$R_{L_{op}}=\omega L_{2}\sqrt{\frac{1}{Q_{2}^{\;\;\;2}}+k^{2}\frac{Q_{1}}{Q_{2}}}\;,$$

where $Q_{i}$ represents the quality factor of the coils (i=1,2) that can be calculated by:

(17) $$Q_{i}=\frac{\omega L_{i}}{R_{i}}\;.$$

The mutual inductance also must be expressed as function of dimensions to fully evaluate Equation (15). Mutual inductance between rectangular coils have no simple analytical formulation, thus the Neumann formula is used for the calculation:

(18) $$M\left(c_{1},c_{2}\right)=\frac{\mu_{r}\mu_{0}}{4\pi }\oint_{c_{1}}\oint_{c_{2}}\frac{\vec{dc_{1}}\cdot \vec{dc_{2}}}{\left|\vec{r_{1}}-\vec{r_{2}}\right|}\;,$$

where $c_{1}$ and $c_{2}$ are the curves that represent the transmitter and the receiver coils, respectively, and $r_{1}$ and $r_{2}$ are the positions of the infinitesimal segments of each coil.

For numerical implementation, the integrals can be discretized as presented in [29]. By solving the equation for two rectangular coils, the transmitter is assumed to be centered at the origin of a cartesian coordinate system and the receiver’s geometric center at the position $\left(x_{RX},y_{RX},z_{RX}\right)$. Therefore, the mutual inductance can be described as function of both coils’ dimensions and the receiver’s position.

#### 3.2.2. Optimization Procedure

Once all elements off the coil’s equivalent circuit are described as a function of the dimensions and receiver’s position, they can be introduced into Equation (15) to evaluate the efficiency as a function of the following parameters:

(19) $$\eta =\eta \left(a_{i},b_{i},w_{i},g_{i},t,N_{i},x_{RX},y_{RX},z_{RX},R_{L}\right)$$

For the optimal efficiency, Equation (16) can be used to calculate the optimal load resistance as function of the dimensions. However, the optimal efficiency is achieved for a given positioning of the receiver and load value. That means, the efficiency changes as the receiver moves or the load changes. At this point, the load is considered to be constant and equal to the optimal value. The goal is then to find the dimensions of the Tx and Rx coils for which the circuit operates with at least a threshold efficiency over an entire surface. By doing so, the free-positioning of the receiver is assured, since wherever it is horizontally placed inside the surface, the system operates at high and quasi-uniform efficiency.

**Figure 5:**
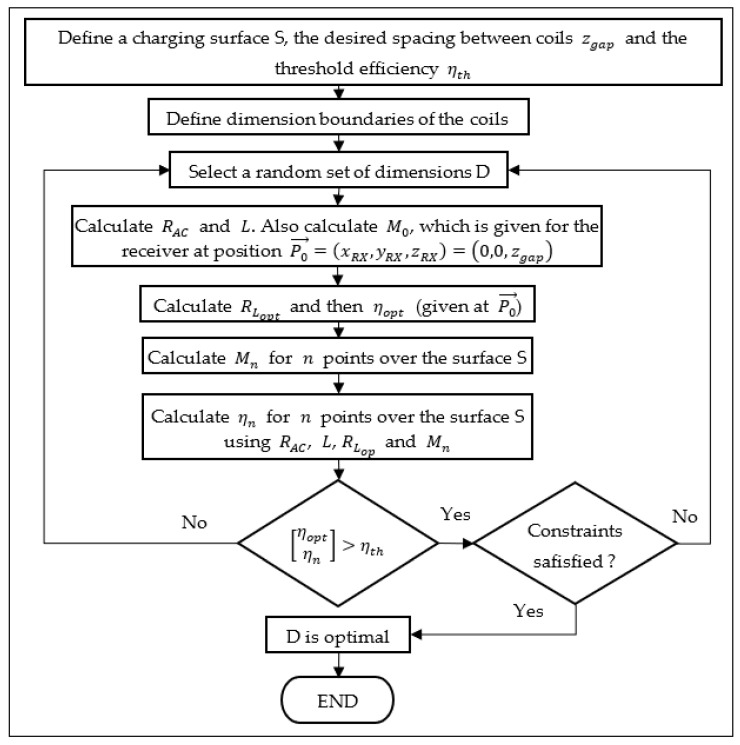
Flowchart of the proposed optimization procedure.

The optimization procedure proposed is described in the flowchart of Figure 5. As it can be seen, the threshold efficiency, the separation between coils, the size of the desired charging surface, and the boundaries of the dimensions are the inputs used to calculate the efficiency of the system for several positions of Rx. The optimal load value is calculated for the case when both Tx and Rx are aligned with the separation $z_{gap}$.

If the efficiency for all positions is greater than the threshold and the constraints are satisfied, the optimal dimensions of the coils are found. Constraints are optional and can be used for defining the minimum and maximal values of the quality factor, inductance, resistance, etc. However, two important constraints that must be considered are the minimal self-resonance frequency of each coil and, for the sake of the algorithm, the internal length and width of the coils must be positive.

The aforementioned procedure can only be executed by a mixed-integer algorithm because the number of turns is restricted to integers. Differential evolution and genetic algorithms are candidates to solve the problem. Therefore, the genetic algorithm functions provided by the global optimization toolbox of MATLAB^®^ 2020b (The MathWorks, Inc., Natick, MA, USA) are suitable options for the purpose of this work.

An optimization using MATLAB^®^ was executed with 13.56 MHz as the operating frequency, with a threshold efficiency of $\eta_{\mathrm{th}}=82\%$, operating separation between coils of $z_{\mathrm{gap}}=5\;\mathrm{mm}$, charging surface of 12 cm ×8 cm, and the dimensions of the receiver limited to 4 cm ×4 cm maximum. The conductor’s width and spacing between turns might vary between 0.3 mm and 1.2 mm, the conductor was set to copper with thickness of 35 μm, and the number of turns was set to a minimum of two. Some constrains were defined, such as a maximum quality factor of 90, maximal inductance of 3 μH, positive internal dimensions, and minimal self-resonance frequency. Table 1 illustrates the dimensions of the coils calculated by the algorithm.

**Figure 6:**
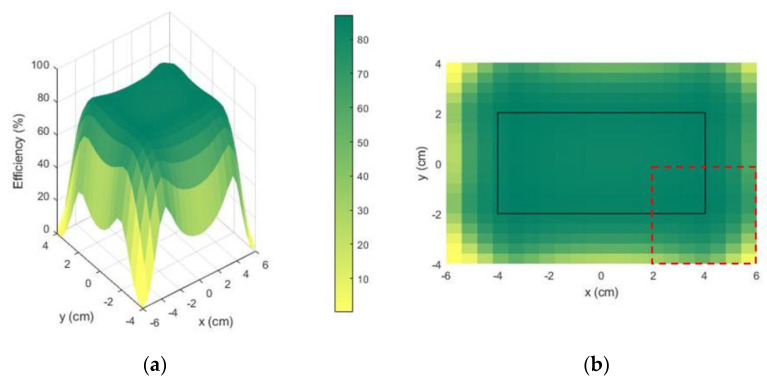
MATLAB^®^ optimization results (efficiency in %) are represented as (**a**) a 3D plot of efficiency vs. the x and y axis; (**b**) 2D plot of the efficiency seen by the top. The coordinates x and y represent the center position of the receiver. The dashed red line represents the Rx contour in an extreme position (its “center” is located at x = 4 cm and y = −2 cm).

Figure 6a illustrates the 3D plot of system’s efficiency when the receiver’s geometric center moves over the x and y axis. A rectangular plateau is formed in the middle of the charging area. It represents the surface on which the center of the receiver can move, the edges of the plateau (better visualized in Figure 6b through the black rectangle) are the limit positions, since at least one edge of the receiver overlays one edge of the charging area.

The rectangular plateau (8 cm ×4 cm) can be understood as the effective charging area, where the geometrical center of the receiver can move. The receiver (4cm ×4 cm) can to move on the surface (12 cm ×8 cm) where the efficiency ranges from 82.2% (on the center) to 87.2% on the corners. The efficiency on the center is not the maxima because of the non-linear behavior of the mutual inductance as a function of the dimensions. However, the output is a system that satisfies the criteria $\eta_{\mathrm{th}}=82\%$, over the entire surface, which does not necessarily mean the center would be the maximum.

**Figure 7:**
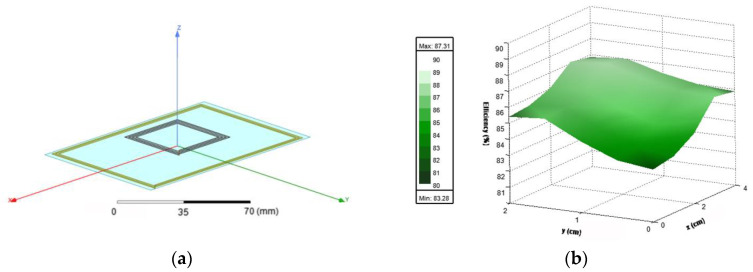
(**a**) 3D Model representing the optimized coils and the representation of the charging surface; (**b**) electromagnetic simulation results of the efficiency (%) of the optimized coils.

Using the electromagnetic simulation software HFSS^®^ (ANSYS, Inc., Canonsburg, PA, USA), the optimized system was modelled (Figure 7a) and the efficiency was simulated. The result is illustrated in Figure 7b, but thanks to the symmetry, instead of simulating the whole surface, only a quadrant is sufficiently representative. The simulated efficiency ranges from 83.3% (on the center) to 87.3%. The simulation results present a minimal discrepancy regarding the theory, which can be justified by the approximations made on (4).

**Figure 8:**
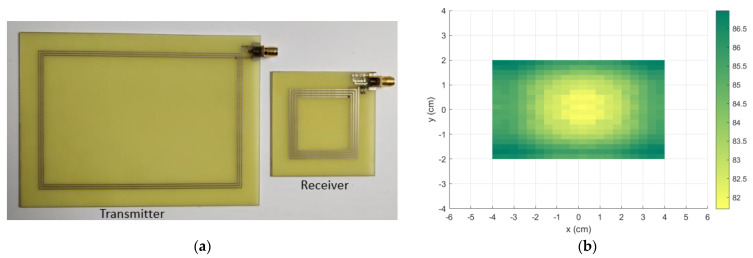
(**a**) The manufactured transmitter and receiver prototypes; (**b**) the experimental efficiency (%) results of the optimized coils for all charging area. Only positions inside the effective charging area were measured.

In order to fully validate the procedure, the optimized coils were prototyped, as can be seen in Figure 8a. The receiver coil was connected to a 104.7 pF capacitor to enable resonance. Using a Vector Network Analyzer (VNA) Keysight E5061B (Keysight Technologies, Inc., Santa Rosa, CA, USA), the S parameters of the coils were measured for different horizonal positions of the receiver. The results were post treated, converting the S parameters to Z parameters and assuming the optimal value of the load resistance. The efficiency for all the measured positions was evaluated and the results are show in Figure 8b. Only positions belonging to the effective charging area were measured.

At last, the electrical parameters were measured using an Agilent E4990A impedance analyzer (Agilent Technologies, Inc., Santa Clara, CA, USA). The results are presented in Table 2, as well as the results from theory and simulations. It also summarizes the maximum and minimum values of efficiency. As can be seen, the experimental results are in good agreement with the theory, presenting a small discrepancy that can be explained by the approximations on resistance and inductance calculation.

## 4. System Optimization Proposition

The optimization of the dimensions presented in last section is a powerful tool to design coils for WPT and NFC. However, the procedure considers a simple circuit composed by a transmitter coil with no impedance matching circuit and a receiver coil with a series resonance capacitor. In addition, the load is assumed to be the optimal for the case when both Tx and Rx are aligned, and variations are not considered. However, a more complete optimization procedure would consider a load that might vary (e.g., a battery). Therefore, additional circuitry must be added to reduce the power losses due to load variation.

The system optimization proposition consists of a three-step procedure. First, the optimization of the dimensions from the last section is simplified to the figure of merit optimization instead of efficiency. Afterwards, the receiver is optimized by analyzing its compensation network configuration. Finally, the third step consists of optimizing the transmitter, with which an impedance matching network is used to compensate at most the variations of the coupling factor and load that could not have been completely managed in Steps 1 and 2. These steps are described in detail in the following.

### 4.1. Optimization of Dimensions through Figure of Merit Maximization

As can be seen in Equation (13), the mutual inductance is proportional to the efficiency and, as consequence, the coupling factor is also proportional to the efficiency. Therefore, in order to simplify the optimization procedure, it is suggested to optimize the coupling factor instead of the efficiency. By doing so, only the mutual inductance and each coil’s inductance must be considered, which simplifies the circuit hypothesis made in Section 3. In other words, the resonance capacitance and the load do not enter in the optimization procedure. In fact, optimizing the coupling factor for all positions of the receiver over the desired surface is a sufficient condition to assure the minimal loss of energy by electromagnetic coupling. However, if the series resistance is not considered in the optimization, the outcome might produce high-resistance coils, which are not desired because heat losses would be important. Therefore, two solutions are possible: to maximize the coupling factor and add a constraint of maximum resistance or a minimum quality factor; or to maximize the figure of merit (Equation (20)), which considers both the coupling factor and quality factor maximization. The second approach was chosen because it is less demanding in terms of calculation time, since no constraints are required.

(20) $$FOM=kQ_{1}Q_{2}\;.$$

**Figure 9:**
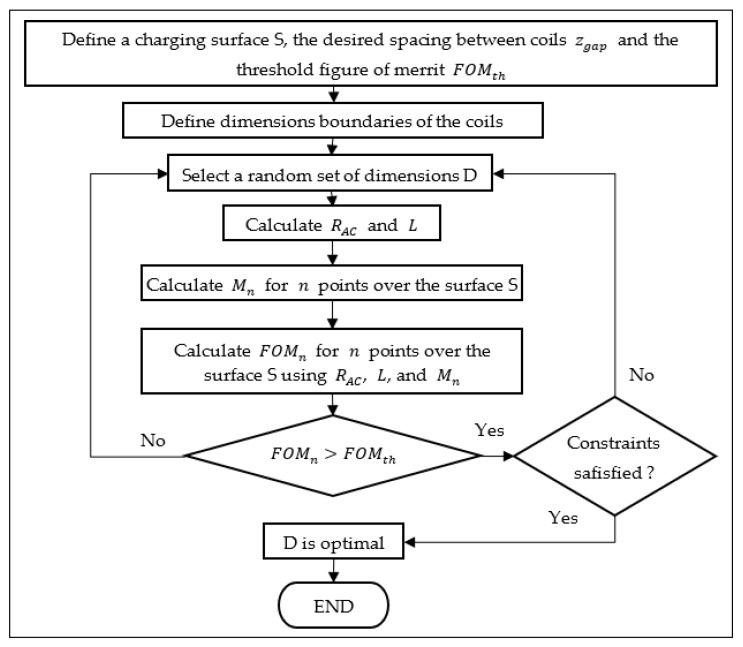
Flowchart of the newly proposed optimization procedure.

By maximizing the FOM, the dimensions are optimized, not only providing the highest magnetic coupling possible (and thus highest coupling efficiency) but also the lowest series resistance, which means the lowest losses by Joule effect. Therefore, the new proposed optimization procedure is presented in Figure 9.

### 4.2. Receiver’s Circuit Design and Optimization

Once the dimensions are optimized, the coupling efficiency is the highest possible for the given couple of Tx and Rx. However, as the load varies, the global efficiency may also vary. In addition, the receiver’s compensation topology affects how the impedance changes. Therefore, it can be designed so the impedance seen by the Tx is not highly affected by elevated load excursions.

As a simplification, a resistance is assumed as the receiver’s total load, which stands for the rectifier and the battery. Three topologies of compensation are analyzed: series capacitor (S), parallel capacitor (P), and series-parallel capacitors (SP). It must be noted that the optimization procedure introduced in Section 3 considered an S topology. For both the S and P configurations, the capacitor is designed so it is in resonance with the coil, whereas the SP compensation provides a degree of freedom that can be used for optimization.

**Figure 10:**
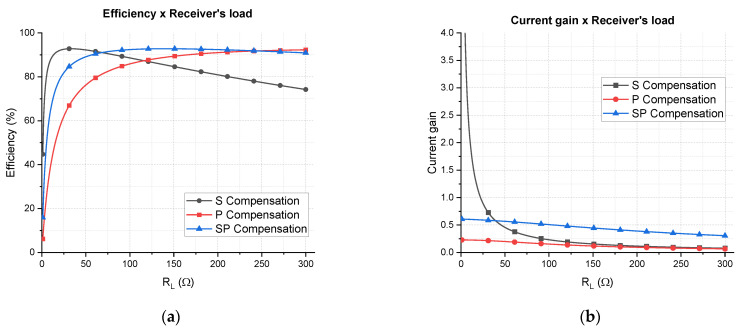
Theoretical results of the S, P, and SP topologies are represented as plots of (**a**) efficiency vs. load resistance; (**b**) current gain (ratio between the current through the load and the current thought the Tx coil) vs. load resistance.

As an example, by using the Tx and Rx coils of Section 3 and supposing a hypothetical coupling factor of 0.15, the efficiency can be calculated as function of the load for the three different compensation networks. The results are shown in Figure 10a, and as can be seen, the S topology is more efficient for low resistances and the P topology is more efficient for high resistances.

The intersection point of the S and P curves depends on the operating frequency, inductance, and load value [30], and can be calculated by the following equation:

(21) $$R_{L_{s-p}}=2\pi fL_{2}\;.$$

For the aforementioned example, Equation (21) gives 110.8 Ω, which is verified in Figure 10a. The SP compensation provides an intermediate behavior, which is better than the P compensation for low resistances and better than S compensation for high resistances. Even though the P compensation is more efficient for very high resistances, WPT systems usually present low and medium load-resistance values. For NFC the contrary is true: since it is a low-power application, the load resistance tends to be higher. However, the objective of this work is to make WPT via NFC, which requires more power than the usual NFC, going from at least 250 mW of delivered power to 1 W. Therefore, the SP compensation draws attention as a promising topology to assure the stability of the efficiency as the load varies.

A special characteristic of the SP compensation network is the flexibility to change the efficiency vs. load curve; that is, as a function of the capacitor values, the curve can be adjusted in between the S and P curves. In fact, if the series capacitor of the SP topology tends to zero and the parallel capacitor tends to the same value used for the P topology, the SP curve would overlay the P curve. Analogously, the opposite is true, which means there are also a set of capacitors that allows the SP curve to overlay the S curve. In between values provide intermediate curves that can be adjusted to better fit an application.

By using a non-linear optimization method, the capacitors of the SP curve represented in Figure 10a were calculated by maximizing the efficiency for all the interval $0<R_{L}<300\;\Omega$. The obtained values are $C_{series}=184\;\mathrm{pF}$ and $C_{parallel}=244\;\mathrm{pF}$.

Figure 10b depicts the current gain; that is, the magnitude of current through the load divided by the current through the Tx coil. It shows that the S topology is highly dependent on the load resistance whereas P and SP are less dependent. It is more desirable to design high current gain WPT systems, since the current required through the transmitter coil would be lower, heating less, and reducing power losses. Using a series capacitor as compensation network, the current gain was elevated for low load resistance, but it decreases fast as the load increases. Both P and SP compensations are more stable, SP being the more advantageous, since its gain is from two to five times greater than the P topology.

The efficiency and current gain analysis show that the SP compensation is the most promising solution regarding load excursion, whereas S compensation is the most performing in current gain for low values of load resistance. The next subsection will design a transmitter considering the effects of each compensation topology in order to conclude about the best compensation network.

### 4.3. Transmitter’s Circuit Design and Optimization

The previous subsection analyzed the three topologies of the compensation network of Rx, illustrating how the SP topology is more interesting than the others for a greater load excursion requirement. However, the analysis is based only on the efficiency and the current gain, which means a further study must be conducted from the transmitter side in order to evaluate how each topology of receiver affects the behavior of the transmitter.

**Figure 11:**
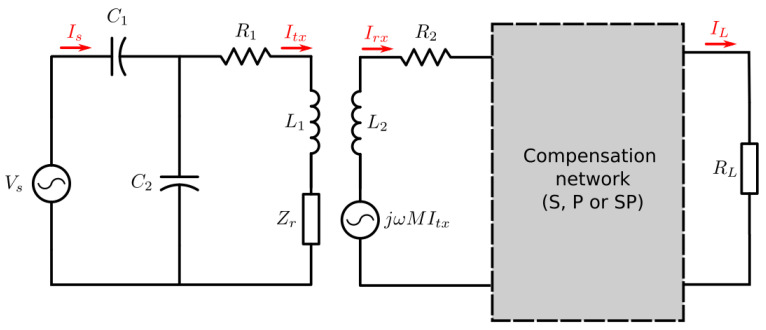
Transmitter and receiver complete circuit.

To do so, the circuit from Figure 11 is considered. A sinusoidal voltage supply $V_{s}$ is connected to an impedance matching network composed of $C_{1}$ and $C_{2}$, which are calculated in order to adapt the transmitter’s impedance to a real value. The impedance $Z_{r}$ is the reflected impedance to the transmitter side, which is proportional to the coupling coefficient between both coils and inversely proportional to the total series impedance of the receiver. Regarding the receiver, a current-dependent voltage source is connected in series with the receiver coil to represent the induced voltage. The coil is then connected to the compensation network and finally to the load.

The capacitors $C_{1}$ and $C_{2}$ are fundamental to assure the condition of maximum power transfer from the supply to the receiver, because they transform the transmitter load impedance to a real value, which prevents the power supply to provide reactive power.

Assuming the coils have a high quality factor (Q>80), the capacitor values can be approximated to Equations (22) and (23).

(22) $$C_{1}\approx \frac{1-\gamma }{\omega X_{L_{p}}}\;\;\;$$

(23) $$C_{2}\approx \frac{\gamma }{\omega X_{L_{p}}}\;\;,$$

where

(24) $$\gamma =1-\sqrt{\frac{R_{p}}{R_{0}}}\;.$$

where $R_{0}$ is the desired matching resistance and $R_{p}$ and $X_{L_{p}}$ are the resistance and reactance seen by the impedance matching circuit, respectively, which can be understood as the transmitter’s load $\left(Z_{p}\right)$ and can be expressed as follows:

(25) $$Z_{p}=R_{p}+j\omega L_{p}=\left(R_{1}+Re\left\{Z_{r}\right\}\right)+j\left(\omega L_{1}+Im\left\{Z_{r}\right\}\right)\;.$$

Due to the nature of the matching network, it must be noted that $Z_{p}$ can only be matched if the following two conditions are satisfied:

(26) $$R_{p}<R_{0}\;,$$

(27) $$X_{L_{p}}=\omega L_{p}>\sqrt{R_{p}\left(R_{0}-R_{p}\right)}\;.$$

Since $R_{p}$ can vary, the minimum value of $X_{L_{p}}$ that allows the matching condition for every $R_{p}$ is defined by $X_{L_{p-critic}}$, which is calculated by taking the derivative of Equation (27) with respect to $R_{p}$ and nulling it, which gives

(28) $$X_{L_{p-critic}}=\frac{R_{0}}{2}\;\;.$$

The value of the capacitors depends on the reflected impedance, which means it depends on the receiver’s load and on the coupling factor. The matching network is usually calculated without the presence of a receiver and then, when a Rx is at the proximity of the Tx, the impedance mismatches, introducing losses to the system.

In order to compensate for the mismatching introduced by a Rx, an automatic impedance matching circuit must be implemented. Practical solutions of such a strategy are difficult to be performed at 13.56 MHz. Digitally tunable capacitors (DTCs), capacitor matrices, and varactors have been studied in the literature [23,24], but DTCs that work properly at high frequency are rare and they only allow a voltage drop of <5 V for the RF signal, which is too small for NFC matching networks. Capacitor matrices need efficient switching devices, which is also difficult to be realized at high frequencies. For instance, two possible solutions for a switch would be FETs and PIN diodes. However, FETs present non-negligible parasitic capacitance and PIN diodes require biasing elements, which demand a big surface for implementation and introduces non-negligible losses. At last, varactors are an interesting approach, but for small signals, which is not the case for WPT. However, there are varactor-based components dedicated for NFC matching networks that support high voltage drops [31]. By using an adaptative matching circuit, the conditions from Equations (26) and (27) can be satisfied even though the load and the coupling factor vary.

**Figure 12:**
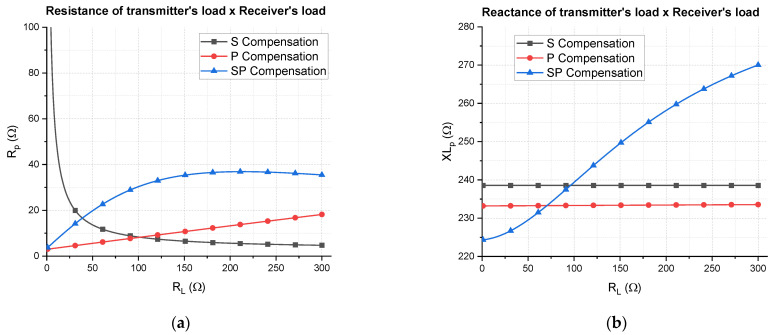
Theoretical transmitter’s load impedance caused by receivers of type S, P, and SP is represented as (**a**) real part vs. receiver’s load; (**b**) imaginary part vs. receiver’s load.

The impact on the load reflected to the transmitter caused by the three types of receiver compensation networks (S, P, and SP) as function of the receiver’s load is depicted in Figure 12. It is an example using the coils designed in Section 3, supposing a coupling factor of 0.15.

Assuming $R_{0}=50\;\Omega$, which gives $X_{L_{p}}>25\;\Omega$ and $R_{p}<50\;\Omega$ from the matching conditions, it is verified that $X_{L_{p}}$ is not a limiting parameter for matching feasibility, but $R_{p}$ imposes restrictions, mainly for the S-type receiver. For $R_{L}<11\;\Omega$, the adaptative matching network is not able to adapt all possible impedances provided from an S-type receiver, meaning that matching losses would be introduced. Therefore, the S-type compensation is not advantageous, since the interval $3\;\Omega <R_{L}<10\;\Omega$ (responsible for a high-efficiency power transfer (70%<η<88%)) cannot be fully exploited.

Regarding the SP-type receiver, there are no restrictions about the matching conditions, since $R_{p_{max}}=36.7\;\Omega$. As for the P type receiver, it would only exceed the $R_{p}$ limit for extremely high values of $R_{L}$. Nevertheless, the P type receiver was already put aside as a solution for NFC WPT in Section 4.2. Even though the SP-type receiver does not impose any restriction regarding the reactance $X_{L_{p}}$, it is the only type for which the reactance varies as a function of $R_{L}$. Therefore, in order to evaluate the impact of this variation, the transmitter’s transconductance is analyzed.

Assuming that the impedance is always matched, thanks to an adaptative matching network, and also that coils have a high quality factor (Q>80), the transconductance of the transmitter can be expressed by the following approximation:

(29) $$g_{m}=\frac{\left|I_{tx}\right|}{\left|V_{s}\right|}\approx \frac{1}{R_{p}\sqrt{1+{\left(\frac{\gamma R_{0}}{\omega L_{p}}\right)}^{2}}}\;$$

**Figure 13:**
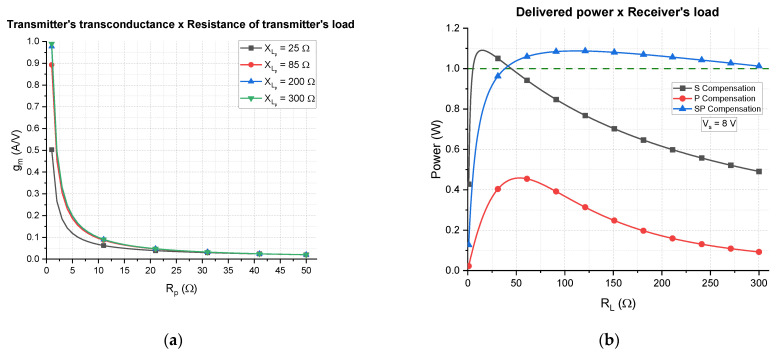
(**a**) Theoretical transmitter’s transconductance vs. resistance of the transmitter’s load for four values of reactance of the transmitter’s load. (**b**) Delivered power to the receiver’s load vs. receiver’s load for the three types of compensation and the sinusoidal voltage supply set to 8 V of amplitude.

From Equation (29), the transconductance can be represented as function of the resistance of the transmitter’s load, as illustrated in Figure 13a. It can be concluded that the transconductance is very sensible to variations in $R_{p}$, especially when $R_{p}<10\;\Omega$, and it is less sensible to $X_{L_{p}}$, especially if it is high; that is, $X_{L_{p}}>85\;\Omega$ (or $L_{p}>1\;\mu H\;$). It means that even though the reactance of the transmitter’s load varies, it still does not affect considerably the transconductance, which can be verified by the fact that the curves of $X_{L_{p}}=200\;\Omega$ and $X_{L_{p}}=300\;\Omega$ overlay each other.

The last remark consists of the analysis of the delivered power to the receiver’s load when the complete Tx-Rx system is considered. The circuit in Figure 11 is supplied by a sinusoidal voltage of peak value $V_{g}=8\;V$ and the power delivered to $R_{L}$ is calculated for the three types of receiver’s compensation network. The results are depicted in Figure 13b, where a threshold of 1 W is represented as dashed line. From this plot, it can be seen that the power delivered to a P-type receiver is the smallest, which reinforces the interest of putting aside the use of this type of compensation network for NFC WPT.

Aiming to deliver 1 W of power, the S and SP compensation networks are candidates. However, compared to the SP type, the S type can deliver 1 W within a smaller interval of load; that is, 6$\;\Omega <R_{L}<44\;\Omega$ for the S type and 39$\;\Omega <R_{L}<300\;\Omega$ for the SP type. In addition, the S-type does not satisfy the matching conditions for $R_{L}<11\;\Omega$. Therefore, considering only the values of load that can be matched, the total load excursion for the S type is $R_{L-exc}=33\;\Omega$, whereas for the SP type it is $R_{L-exc}=261\;\Omega$, which is almost eight times bigger.

By putting together all results from Figure 10, Figure 12 and Figure 13, for these load intervals, it can be concluded that the S type operates at a maximum efficiency of η>88.7% with current amplitude of $I_{TX}<528\;\mathrm{mA}$, whereas the SP-type operates at a maximum efficiency of η>87.0% with $I_{TX}<496\;\mathrm{mA}$. The current levels are important for emission analysis and human exposure assessment, which are not within the scope of this work but is indeed considered in fashion right now as lower current levels are more contemplated.

From the previous analysis, the SP type turns out to be the more interesting compensation network because it presents the greatest load excursion while assuring the desired delivered power and lower current level than the S type. The SP type also holds the matching conditions for all load excursion and regarding the efficiency, even though there are few values of $R_{L}$ for which the S type is more efficient than the SP type; the difference is minimum and the overall advantages of the SP type makes it the best compensation network for NFC WPT purpose. Nevertheless, hybrid topologies can also be used, such as T or π. For further complexity, a T topology could be implemented with a switching device connected to the shunt element: if the switch is open, the topology becomes an S type and if the switch is closed it becomes a T type, which behaves similarly to an SP type. Therefore, the system can operate switching from S type to SP type (and vice versa) as a function of the load value and use the advantages of each compensation network.

## 5. Experimental Results

The first step for designing an optimized pair of WPT coils is to use the optimization method proposed in Section 4.1. By keeping the same order of magnitude of the dimensions of the coils designed in Section 3.2.2, the algorithm was implemented, and the obtained coils were prototyped. The dimensions of the obtained coils are shown in Table 3 and the theoretical, simulation, and experimentation results of the electrical parameters and quality factor are shown in Table 4.

As can be seen in Table 4, results between the simulation and measurements are in good agreement. Some disparities are present and can be justified by the tinning solution added to the prototypes, the accuracy of the measurements, and also the effect of the extra tracks used for making the connection between the coil and the connector. In addition, regarding the theory, it must not be forgotten that some equation approximations were made in order to simplify the calculation. For instance, simplifications do not consider some more complex effects, such as the proximity effects of conductors or border effects. Nevertheless, the results are relatively in good agreement and they are already sufficient to prove the interest of the proposed optimization procedure.

The second step consists of calculating the SP compensation network of the Rx considering the experimental values of the electrical parameters. To do so, a non-linear optimization method was used for maximizing the efficiency for the interval $0<R_{L}<300\;\Omega$. The separation between coils $(z_{gap}$) was set to 10 mm, which is two times bigger than the prototype designed in Section 3.2.2. The obtained values for the compensation network are $C_{series}=142\;\mathrm{pF}$ and $C_{parallel}=285\;\mathrm{pF}$.

Regarding the impedance matching network of the Tx, a varactor-based device such as the one in [31] could be used but due to the need of adding a control circuit and voltage, current, and phase sensors, it was chosen to use trimmer capacitors that can be manually adjusted. In addition, the objective of this work is to design a WPT system regarding energy transfer optimization, which means it is not focused on designing a control system. Nevertheless, it can still be treated as future work, exploring an automatic controlled network with a varactor-based device.

In order to choose the trimmer capacitor, a range of capacitance must be estimated to allow the system to be adapted regardless the impact of the load variation and the coupling factor variation. Maximum and minimum values of load can be extracted from the impedance of the transmitter’s load versus receiver’s load curve, as exemplified in Figure 12. The coupling factor for all positions over the charging surface can also be estimated in theory, and then its maximum and minimum values can be extracted. Therefore, in order to assure the matching conditions, the capacitors values for the impedance matching network must be in the intervals $11\;\mathrm{pF}<C_{1}<39\;\mathrm{pF}$ and $9\;pF<C_{2}<45\;\mathrm{pF}$. For each capacitor, two trimmers of 10 pF<C<100 pF were put in series so they can vary the capacitance from 5 pF to 50 pF, which is sufficient for the designed system.

In practice it is rather difficult to implement a precision varying load resistance in AC, because potentiometers introduce parasitic inductive and capacitive effects that are not negligible at 13.56 MHz. That means, it would not be representative of a real-use case of a bridge rectifier, DC–DC converter, and battery. Therefore, the S parameters of the system are measured for each position of the receiver and then the data are post-processed to calculate the efficiency.

**Figure 14:**
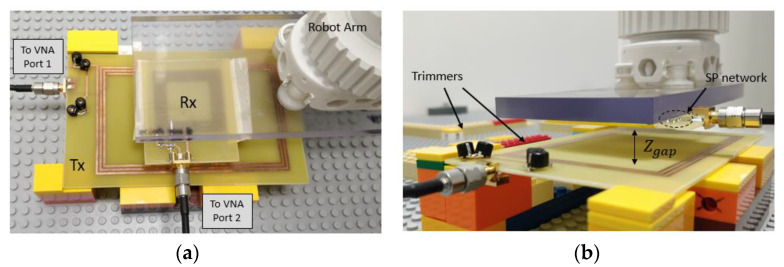
Measurement setup for the new prototypes are shown: (**a**) top view: receiver at horizonal position with VNA connections; (**b**) side view: separation $\left(Z_{gap}=10\;\mathrm{mm}\right)$, trimmers, and SP compensation network.

The measurement setup for the new prototypes are shown in Figure 14. As in Section 3.2.2, a VNA is connected to the Tx and the Rx. The Tx coil is connected to the trimmers and then to the port 1 of the VNA and the Rx coil is connected to the SP compensation network and then to the port 2 of the VNA. The Rx can move thanks to a robotic arm and for each position, the S parameters can be retrieved from the VNA.

**Figure 15:**
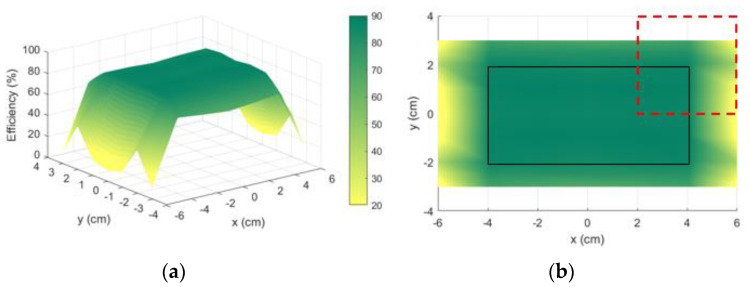
Efficiency measurements for a fixed load of 50 Ω with the input always adapted to 50 Ω: (**a**) 3D plot of efficiency vs. the x and y axes; (**b**) 2D plot of the efficiency seen from the top. The coordinates x and y represent the center position of the receiver. The dashed line represents the Rx contour in an extreme position (its “center” is located at x = 4 cm and y = −2 cm).

The first measurement consisted of representing the efficiency of the system for all possible positions of the Rx. To do so, the Rx move over the charging surface along the x and y axis with a separation of $z_{\mathrm{gap}}=10\;\mathrm{mm}$. For each position, the trimmers are adjusted in order obtain an input impedance of 50 Ω at the transmitter side. Since the Rx is connected to the VNA, it is considered that the Rx is connected to a load of 50 Ω. The results are represented in Figure 15 as a 3D plot and a 2D plot of the efficiency. In the 2D plot, the solid rectangle represents the maximum position that the geometric center of the Rx can achieve without leaving the charging surface (8 cm ×12 cm). The dashed line represents the receiver at a corner position. For all positions of Rx, the system is capable to operate with at least 84.8%. It performs better than the previous prototype developed in Section 3.2.2, for which the minimum efficiency was only 81.7%, even with the optimal load. Besides, the separation between coils were half that of the actual prototype, this being 5 mm.

**Figure 16:**
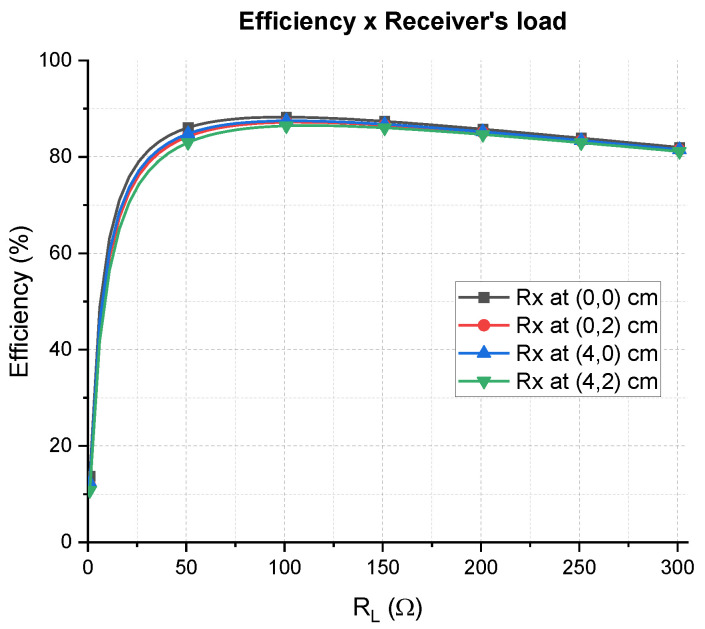
Efficiency vs. load resistance for four different positions of the receiver. The Rx is connected to an SP compensation network and the Tx is always adapted to 50 Ω.

In order to evaluate the effects of the load variation, four positions of Rx were considered: the center (0,0), a corner (4 cm,2 cm), a middle point over the length (0, 2 cm), and a middle point over the width (4 cm,0). For each position, the S parameters of the system were used to calculate the efficiency considering load variation. The results are represented in Figure 16. As can be seen, even for the worst-case position, which is the corner, the efficiency of the system is held above 80% for the interval $36\;\Omega <R_{L}<300\;\Omega$, which means a 264 Ω load excursion. The center is the best case, for which the efficiency is also held above 80%, but the load excursion is bigger (274 Ω), with an interval of $26\;\Omega <R_{L}<300\;\Omega$.

## 6. Conclusions

This paper provided a new optimization procedure (regarding a prior study [16]) for designing an efficient WPT system operating at 13.56 MHz, dedicated to the new trending NFC wireless charging technology. The system is developed in such a way that it assures a 2D free-positioning of the receiver and low sensibility to load variation.

A theoretical background was developed for the optimization procedure, which calculates the optimal dimensions of a pair of transmitter and receiver rectangular-shaped coils in order to assure the highest efficiency for all positions of a receiver over a surface. The main advantage of the proposed procedure is to facilitate the design of coils, since the most common approach is to simulate several coils of different dimensions with electromagnetic simulation software, which can be time consuming. The proposed procedure seeks the automation of the coil design, providing a highly efficient pair of coils according to the dimensional requirements. The coils are optimized in a fashion that the receiver can be freely positioned on a surface; that is, regardless the its position, the system is able to operate at a high efficiency.

In addition to the optimization procedure, the compensation network of the receiver was analyzed, and the SP topology is proposed as the most promising for NFC wireless power transfer. This topology allows the system to operate at a high efficiency, for a great load excursion and also to deliver power levels that are in accordance with the new NFC WLC technical specification [10]. Therefore, the study realized in this work can be used as guidelines for designing coils for devices that seek to be compliant to the NFC Forum wireless power transfer specification.

Furthermore, a circuit analysis of the transmitter input impedance simplifies equations that can be used for future work, such as the implementation of the control algorithm for automatic impedance matching using a proposed varactor-based device. Even though this work does not implement an automatic impedance matching, trimmers are used to show the relevance of the results.

With the prior-developed optimization procedure, a transmitter (7.7 cm ×11.3 cm) and receiver (4 cm ×4 cm) pair was prototyped. Considering a separation of 5 mm between the coils and the load fixed to the optimum value, the system operates with efficiency between 81.7% and 87% over a 8 cm×12 cm charging surface.

With the new optimization method and introducing the circuit optimizations (SP topology and automatic impedance matching network), a new pair of coils were calculated and prototyped. The same order of magnitude of the prior procedure’s coil dimensions and charging surface was kept, but the separation was doubled to 10 mm. The results were a transmitter of 7 cm×11.4 cm and a receiver of 4 cm ×4 cm. The new system operates above 80%, reaching up to 88.3% for a load interval of $36\;\Omega <R_{L}<300\;\Omega$, which means 274 Ω of load excursion. Therefore, the system operates at high efficiency across the whole charging area and presents a very low sensibility to load variations, which can be very promising for battery-charging applications. Regarding the power, for the load interval aforementioned, the obtained system is able to deliver at least 1 W to the receiver, assuming a sinusoidal input voltage of 8 V of the peak value.

In a nutshell, this work proposes an optimization method for printed rectangular-shaped coils and for multiple positions of a receiver, whereas the state-of-the-art method proposes only optimizations for printed squared-shaped coils and for a single position of a receiver. In addition, in the frame of WPT via NFC, a compensation network of the receiver was proposed in order to assure a high efficiency with low load sensibility.

## Figures and Tables

**Table 1:** Dimensions of the obtained coils using the proposed efficiency optimization method.

	N	a (cm)	b (cm)	w (mm)	g (mm)
Tx	3	11.27	7.65	0.3	0.9
Rx	4	4	4	0.4	1

**Table 2:** Comparative table of the theoretical, simulation, and experimental results of the efficiency and electrical parameters at 13.56 MHz.

	$\eta_{min}$	$\eta_{max}$	$R_{eq}\;(\mathrm{Tx})$	$L_{eq}\;(\mathrm{Tx})$	$R_{eq}\;(\mathrm{Rx})$	$L_{eq}\;(\mathrm{Rx})$
Theory	82.2%	87.2%	2.93 Ω	2.83 µH	1.17 Ω	1.24 µH
Simulation	83.3%	87.3%	2.78 Ω	2.88 µH	1.07 Ω	1.27 µH
Measurements	81.7%	87.0%	2.88 Ω	2.99 µH	1.23 Ω	1.30 µH

**Table 3:** Dimensions of the obtained coils using the proposed FOM optimization method.

	N	a (cm)	b (cm)	w (mm)	g (mm)
Tx	3	11.4	7	1.2	0.9
Rx	5	4	4	1.2	0.4

**Table 4:** Comparative table of theoretical results, simulations, and measurements of the electrical parameters and quality factor at 13.56 MHz.

	$R_{eq}\;(\mathrm{Tx})$	$L_{eq}\;(\mathrm{Tx})$	Q (Tx)	$R_{eq}\;(\mathrm{Rx})$	$L_{eq}\;(\mathrm{Rx})$	Q (Rx)
Theory	846 mΩ	2.08 µH	210	527 mΩ	1.36 µH	220
Simulations	836 mΩ	2.17 µH	221	628 mΩ	1.40 µH	190
Measurements	978 mΩ	2.33 µH	202	663 mΩ	1.41 µH	181
